# LGFC-CNN: Prediction of lncRNA-Protein Interactions by Using Multiple Types of Features through Deep Learning

**DOI:** 10.3390/genes12111689

**Published:** 2021-10-24

**Authors:** Lan Huang, Shaoqing Jiao, Sen Yang, Shuangquan Zhang, Xiaopeng Zhu, Rui Guo, Yan Wang

**Affiliations:** 1 Key Laboratory of Symbolic Computation and Knowledge Engineering, Ministry of Education, College of Computer Science and Technology, Jilin University, Changchun 130012, China; huanglan@jlu.edu.cn (L.H.); jiaosq19@mails.jlu.edu.cn (S.J.); shuangquan18@mails.jlu.edu.cn (S.Z.); zhuxiaopen11@163.com (X.Z.); sdsbshe@163.com (R.G.); 2 College of Software, Jilin University, Changchun 130012, China; 3 School of Computer Science and Artificial Intelligence & Aliyun School of Big Data, Changzhou University, Changzhou 213164, China; ystop2020@gmail.com; 4 College of Artificial Intelligence, Jilin University, Changchun 130012, China

**Keywords:** lncRNA-protein interactions, convolutional neural network, two sequence preprocessing methods, raw sequence features, hand-designed features, structure features

## Abstract

Long noncoding RNA (lncRNA) plays a crucial role in many critical biological processes and participates in complex human diseases through interaction with proteins. Considering that identifying lncRNA–protein interactions through experimental methods is expensive and time-consuming, we propose a novel method based on deep learning that combines raw sequence composition features, hand-designed features and structure features, called LGFC-CNN, to predict lncRNA–protein interactions. The two sequence preprocessing methods and CNN modules (GloCNN and LocCNN) are utilized to extract the raw sequence global and local features. Meanwhile, we select hand-designed features by comparing the predictive effect of different lncRNA and protein features combinations. Furthermore, we obtain the structure features and unifying the dimensions through Fourier transform. In the end, the four types of features are integrated to comprehensively predict the lncRNA–protein interactions. Compared with other state-of-the-art methods on three lncRNA–protein interaction datasets, LGFC-CNN achieves the best performance with an accuracy of 94.14%, on RPI21850; an accuracy of 92.94%, on RPI7317; and an accuracy of 98.19% on RPI1847. The results show that our LGFC-CNN can effectively predict the lncRNA–protein interactions by combining raw sequence composition features, hand-designed features and structure features.

## 1. Introduction

Long noncoding RNA (lncRNA) is a type of noncoding RNA with at least 200 nucleotides that plays vital roles in many critical biological processes [1], such as cell differentiation, gene expression, and the display of developmental and tissue-specific expression patterns [2,3,4]. Some lncRNA regulates a wide range of biological processes through interactions with miRNAs. They compete with mRNA for binding to the same miRNA to create a competitive endogenous RNA (ceRNA) regulatory network through which their modular structure permits their interaction with miRNAs [5,6]. Some lncRNA participates in many complex human diseases by interacting with proteins [7]. For example, silencing lncRNA-5657 inhibits the pneumonia lung inflammatory response via suppressing the expression of spinster homology protein2, thereby reducing sepsis-induced lung injury [8]. FAM83H-AS1 contributes to radioresistance and cell metastasis in ovarian cancer through the stabilizing HuR protein [9]. LncRNA NEAT1 promotes MPTP-induced autophagy in Parkinson’s disease through the stabilization of the PINK1 protein [10]. Therefore, predicting potential lncRNA–protein interactions is a crucial step in understanding the function of lncRNA and creating the conditions for solving complex human diseases. With the development of experimental technology, computational methods have become crucial as a silver-bullet solution for the large-scale capture of lncRNA–protein interactions, which helps to prioritize lncRNA–protein interaction candidates and conduct further experimental verification. 

Existing computational methods can be categorized into network-based methods and machine learning-based methods. Network-based methods construct a lncRNA/protein similarity matrix and then use network algorithms to calculate correlation scores to make predictions. For example, Zhang et al. proposed a model called LPLNP, in 2017, which calculated the linear neighborhood similarity between lncRNAs and proteins and the regularized linear neighborhood similarity and predicted the observed lncRNA–protein interactions by a label-propagation process [11]. Zhao et al. introduced a method named LPI-BNPRA in 2018, which used the known lncRNA–protein interactions matrix, lncRNA similarity matrix, and protein similarity matrix to predict lncRNA–protein relationships [12]. Zhu et al. presented a model named ACCBN, in 2019, the model first to use an ant-colony algorithm for data clustering and then constructed a lncRNA–protein bipartite network inference (LPBNI) to predict lncRNA–protein interactions [13]. However, network-based methods require that each node in the network has at least two linkages; the lncRNA–protein interaction network is composed of a few isolated subnetworks, and the imbalance of the degree distribution of each node in the network will also affect its prediction performance [14].

The machine learning-based methods extract manual features from lncRNA and protein sequences to represent lncRNA–protein pairs and then input them into machine learning classifiers to predict lncRNA–protein interaction pairs. For example, Ge et al. proposed a model named RPISeq, in 2015, that input the 4-mer frequency characteristics of RNA sequences and the 3-mer frequency characteristics of proteins into a random forest classifier and support-vector-machine classifiers to identify RNA-protein interactions [15]. Pan et al. developed a method called IPMiner, in 2016, that input raw sequence-composition features, the advanced features extracted by a cascaded autoencoder and the features extracted by fine-tuned cascaded noise reduction auto-encoding into a random forest classifier, then used a cascading ensemble to integrate the output of the above three classifiers to predict lncRNA–protein interactions [16]. In 2019, Fan et al. proposed LPI-BLS; they first combined lncRNA’s and protein’s features, input these features into five separate extensive learning systems, and finally integrated separate BLS classifiers through a stacking integration strategy to obtain their prediction results [17]. Liu et al. presented a model named LPI-NRLMF in 2017, which mapped the lncRNA–protein interaction matrix to the lncRNA similarity matrix and the protein similarity matrix to predict the possibility of lncRNA–protein interactions [18]. However, the machine learning-based methods have limitations that rely on the quality of hand-designed features [19,20]. 

In this paper, we propose a new deep-learning model (LGFC-CNN) that combines raw sequence-composition features, hand-designed features, and structure features to comprehensively predict lncRNA–protein interactions. First, we improve the sequences’ preprocessing, originally used to predict RNA-protein binding sites, and apply it to transform the sequences into fixed-length sequences [21]. After that, the lncRNA and protein sequences are encoded by using one-hot encoding [22,23] and fed into GloCNN and LocCNN modules to extract the raw sequence’s global and local features. Meanwhile, a random forest (RF) classifier [24] is employed to compare various lncRNA’s and protein’s hand-designed combinations of features, and, of such features, those with the three most-superior predictive effects are fed into an FC module to gain useful information. Furthermore, the secondary structures, hydrogen bonding, and van der Waals interactions of the lncRNA and protein are encoded and fed into an SS module, after unifying their feature dimensions through a Fourier transform. Finally, the four network modules are integrated, to improve predictive performance by analyzing multiple types of features. In addition, comparing LGFC-CNN with several existing methods, the results show that LGFC-CNN is a competitive method for effectively predicting lncRNA–protein interactions.

## 2. Materials and Methods

**Figure 1:**
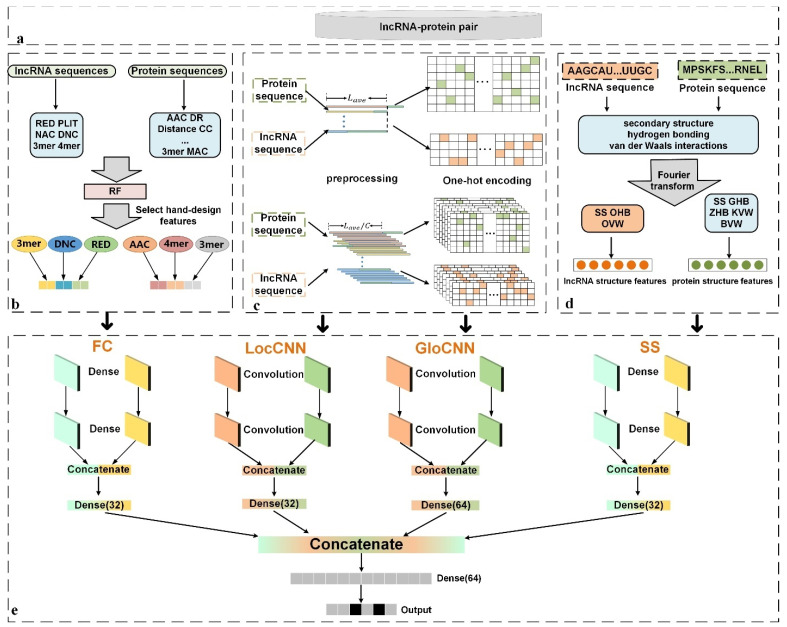
The flowchart of LPI-CNNCP. (**a**) Build lncRNA-protein interactions datasets and obtain lncRNA and protein sequences; (**b**) Feed lncRNA and protein hand-designed feature combinations into RF classifier to select the hand-designed features with superior predictive effect; (**c**) lncRNA and protein sequences are preprocessed by two methods and encoded by using one-hot encoding; (**d**) lncRNA and protein secondary structure, hydrogen bonding propensities, and van der Waals interactions are obtained and unifying the dimensions through Fourier transform. (e) Feed the global and local encoded sequences, hand-designed features and structure features into CNN model to predict the lncRNA-protein interactions.

An illustration of LGFC-CNN for predicting lncRNA–protein interactions is shown in Figure 1.

### 2.1. Construction of Datasets

To evaluate the performance of LGFC-CNN, we test it on the lncRNA–protein interaction datasets of $D_{RPI21850}=D^{+}\cup D^{-}$ (named RPI21850) constructed from the NPInterv4.0 database [25,26]. To filter lncRNAs and their interacting proteins, the ncRNA sequences whose length less than 200nt and the lncRNA–protein interactions not from Homo sapiens were excluded. Then, we constructed a positive dataset $D^{+}$, which contained 21850 pairs of high-confidence lncRNA–protein interactions consisting of 4221 lncRNAs and 701 proteins.

Due to the lack of negative samples in the NPInter4.0 database and the assumption that obtaining the negative dataset by randomly pairing lncRNA and protein is not entirely reasonable, we adopted the following criteria from FIRE [27] to build the high-quality negative dataset $D^{-}$: 

For a lncRNA–protein interaction of protein $p_{1}$ and RNA r, r is highly possible to interact with any protein, $p_{2}$, similar to $p_{1}$. Contrarily, if protein $p_{2}$ is dissimilar to $p_{1}$, there is a low possibility that $p_{2}$ interacts r [27]. Therefore, we used the pairwise2 module in Biopython [28] to calculate the global sequence similarity score *S_s_* between all proteins from the positive dataset. Then, we sorted the global sequence similarity scores Ss between the proteins, in ascending order.

**Figure 2:**
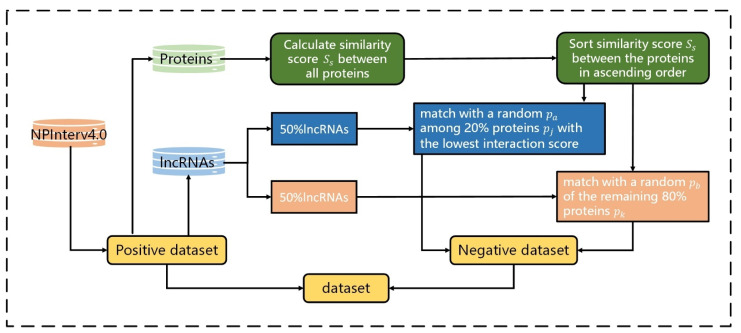
The flowchart of constructing reliable negative samples. The positive dataset is constructed by extracting interactions with NPInterv4.0. We calculated and sorted the similarity scores, *S_s_*, between all proteins from the positive dataset, and divided all lncRNAs into two equal parts before applying different strategies to build the negative dataset.

To reduce the repeated lncRNA–protein interactions and consider that lncRNA has a certain probability of having a relationship with those proteins with higher scores, instead of selecting the lncRNA–protein pairs with the lowest interaction scores, we divided all lncRNAs into two equal parts. In the first part, the lncRNA–protein pairs were selected for which their lncRNA matched with a random $p_{a}$ among the 20% of proteins, $p_{j}$, with the lowest interaction scores. In the second part, the lncRNA–protein pairs were selected for which their lncRNA matched with a random $p_{b}$ of the remaining 80% of proteins, $p_{k}$. Then, the two parts were combined to build a negative dataset, $D^{-}$, that containe 21850 lncRNA–protein pairs. The flowchart of constructing reliable negative samples is shown in Figure 2.

To further assess the reliability and robustness of LGFC-CNN, RPI7317 and RPI1847 in LPI-BLS [16] were constructed by adopting a similar method as in assessing RPI21850, and the numbers of lncRNA–protein interacting pairs they contained were 7317 and 1847, respectively. There was no overlap between RPI7317, PRI1847, and RPI21850. The corresponding lncRNA sequences were obtained by NONECODE v6.0 [29], and the corresponding protein sequences were obtained by UniProt [30]. Table 1 lists the numeric description of the datasets.

### 2.2. Sequence Encoding

As the CNN model requires fixed-length sequence inputs, whereas different lncRNA(or protein) sequences vary significantly in their lengths, we improved the sequences preprocessing in iDeepE [21] to transform the sequences into the fixed-length sequences. Considering that some lncRNA sequences are extremely long (more than 80,000 bp), we set the average sequence length, $L_{lnc}$ and $L_{pro}$, to represent the lncRNA and protein sequences’ fixed lengths, respectively. Since the local structure of a sequence allows us to understand its protein–RNA binding nature, in terms of structural fragments [31]—which can supplement the lack of global structure—we performed two preprocessing procedures on the raw sequence.

For the GloCNN module, if the lncRNA sequence length was greater than $L_{lnc}$, the sequence was cropped to the fixed length; when lesser, it was extended to the fixed length with nucleotide N.

For the LocCNN module, a lncRNA sequence was divided into subsequences of W windows, in which each subsequence is regarded as a channel and where each window has S overlapping shifts; the size of each window was $L_{lnc}/W$. Here, we calculated the maximum number of channels, *C*, according to the sequence length. If the number of channels for one sequence was greater than C, the sequence was cropped to C.; when lesser, it was extended by channels derived from sequences with all nucleotide *N*s to *C*. 

For a given protein sequence, we adopted the same preprocessing to transform it into the fixed-length sequence with $L_{pro}$. After that, the lncRNA and protein sequences were encoded by using one-hot encoding [22,23]. Given a lncRNA sequence $S=\left(s_{1},s_{2},\ldots ,s_{n}\right)$ with $L_{lnc}$ nucleotides, conversion of the matrix, M, by one-hot encoding, can be expressed as [21]:

(1) $$M_{i,j}=\{\begin{array}{l} 1/4\;if\;s_{i-m+1}=N\;or\;i\langle m\;or\;i\rangle n-m\\ 1\;if\;s_{i-m+1}\;in\;\left\{A,C,G,U\right\}\\ 0\;otherwise \end{array}$$

where i is the index of nucleotide,  j is the index of A,C,G,U in the matrix *M*. For the padded nucleotide at the start and end of sequences, we assumed four nucleotides were equally distributed. Thus, [0.25, 0.25, 0.25, 0.25] was used in the padded nucleotides and N in the one-hot matrix.

**Figure 3:**
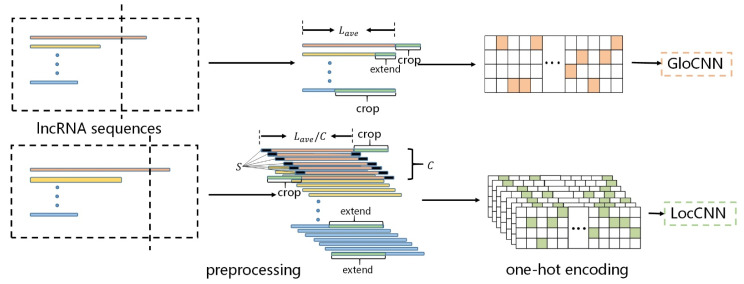
The flowchart of lncRNA sequence encoding. The two preprocessing methods are applied to transform the lncRNA sequences into fixed-length sequences. After that, the sequences are encoded by using one-hot encoding and fed into GloCNN and LocCNN. The protein sequences apply the same sequence encoding.

For a given protein sequence $P=\left(p_{1},p_{2},\ldots ,p_{n}\right)$ with $L_{pro}$ amino acids, the sequence is composed of 20 natural amino acids (*A*, *C*, *D*, *E*, *F*, *G*, *H*, *I*, *K*, *L*, *M*, *N*, *P*, *Q*, *R*, *S*, *T*, *V*, *W*, *Y*). When using one-hot encoding to encode the protein sequence, the encoding matrix can be vast and sparse. Thus, we compressed the 20 amino acid alphabets into seven groups, based on their dipole moments and side chains [32]: $R_{1}=\left\{A,G,V\right\}$, $R_{2}=\left\{I,L,F,P\right\}$, $R_{3}=\left\{Y,M,T,S\right\}$, $R_{4}=\left\{H,N,Q,W\right\}$, $R_{5}=\left\{R,K\right\}$, $R_{6}=\left\{D,E\right\}$, $R_{7}=\left\{C\right\}$. The matrix *R*, converted by one-hot encoding, can be expressed as:

(2) $$R_{i,j}=\{\begin{array}{l} 1/7if\;p_{i-m+1}=N\;or\;i\langle m\;or\;i\rangle n-m\\ 1if\;p_{i-m+1}\;in\;\left\{R_{1},R_{2},R_{3},R_{4},R_{5},R_{6},R_{7}\right\}\\ 0\;otherwise \end{array}$$

where i is the index of the amino acid, j is the index of $R_{1},R_{2},\ldots ,R_{7}$ in matrix R. For the padded at the start and end of sequences, we assume 7 groups are equally distributed. Thus, [1/7,…,1/7] for the padded amino acid and N in the one-hot matrix. The flowchart of lncRNA sequence encoding is shown in Figure 3.

### 2.3. Hand-Designed Features 

In this work, six hand-designed features of lncRNA are combined with ten hand-designed features of the protein. Each feature combination is ranked according to their average performance in the random forest classifier. Then the top three features with superior predictive effect were selected to represent the lncRNA and protein, respectively. For the lncRNA, the top three features were RNA-coding potential characteristics, $L_{RED}$, di-nucleotide composition, $L_{DNC}$, and lncRNA 3-mer frequency, $L_{3mer}$. For the protein, the top three features were amino acid composition, $P_{AAC}$, protein 3-mer frequency, $P_{3mer}$, and protein 4-mer frequency, $P_{4mer}$. For lncRNA–protein pairs, we concatenated the three lncRNA and protein feature vectors to form two feature vectors,$\;A_{1}=\left[L_{RED},L_{DNC},L_{3mer}\right]$,$\;A_{2}=\left[P_{AAC},P_{3mer},P_{4mer}\right]$. The following subsections explain the six feature encodings we used (other feature encodings are explained in Supplementary File S1).

#### 2.3.1. lncRNA Feature RED

CPPred is a tool developed by Xiaoxue Tong et al. to predict coding potential based on the global description of an RNA sequence [33]. It is based on SVM to distinguish ncRNAs from coding RNAs using sequence features, such as ORF length, ORF coverage, ORF integrity, Fickett score, Hexamer score, PI, Gravy, Instability index, and CTD features. Therefore, we use $L_{RED}$ to represent the features generated by CPPred.

(3) $$L_{RED}=\left[ORF-integrity,ORF-civerage,Instability,\ldots ,CTD\right]$$

#### 2.3.2. lncRNA Feature DNC

$L_{DNC}$ describes the A, G, C, and T to represent the trinucleotides by generating a 16-dimensional vector [34,35]. *L*_*DNC*_ can reflect the chemical properties of the accumulated energy of di-nucleotide and reflect the evolutionary information of lncRNA sequences. It can be computed as follows:

(4) $$L_{DNC}\left(r,s\right)=\frac{N_{rs}}{N-1}$$

where $N_{rs}$ is the number of di-nucleotide represented by nucleic acid types *r* and s, N is the length of a nucleotide sequence.

#### 2.3.3. lncRNA Feature 3-mer

$L_{3mer}$ represents the normalized occurrence frequencies of three neighboring base pairs in the RNA sequence [36], which has been successfully applied to human gene regulatory sequence prediction and enhancer identification [37]. It can be computed as follows:

(5) $$L_{3mer}\left(t\right)=\frac{M\left(t\right)}{N},t\in \left\{AAA,AAT,AAC,\ldots ,GGG\right\}$$

where M(t) is the number of k-mer type t, N is the length of a nucleotide sequence.

#### 2.3.4. Protein Feature AAC

The protein sequence is composed of 20 kinds of amino acids. *P*_*ACC*_ provides information regarding the percentage of each residue present in the protein [38]. *P*_*ACC*_ can measure the correlation of two properties or the same properties (hydrophobicity, hydrophilicity, van der Waals normalized volume, polarity etc.) along the protein sequence and convert the matrix to a fixed-length vector [34,39]. It can be computed as follows:

(6) $$f\left(t\right)=\frac{N\left(t\right)}{N}*100$$

where N(t) is the number of amino acids type *t*, N is the length of the protein sequence.

#### 2.3.5. Protein Features 3-mer and 4-mer

For $P_{kmer}$, amino acids are divided into seven groups according to the dipole moment and side-chain volume of the protein [32]: $R_{1}=\left\{A,G,V\right\}$, *R*_2_ = {*I*, *L*, *F*, *P*}, $R_{3}=\left\{Y,M,T,S\right\}$, $R_{4}=\left\{H,N,Q,W\right\}$, $R_{5}=\left\{R,K\right\}$, $R_{6}=\left\{D,E\right\}$, $R_{7}=\left\{C\right\}$. Then, $P_{3mer}$ (the frequency of occurrence of three adjacent coincidences in the protein sequence) and $P_{4mer}$ (the frequency of occurrence of four adjacent symbols in the protein sequence) are obtained. It can be computed as follows:

(7) $$fP_{3mer}\left(t\right)=\frac{Q\left(t\right)}{N},t\in \left\{R_{1}R_{1}R_{1},R_{1}R_{1}R_{2},\ldots ,R_{7}R_{7}R_{7}\right\}$$

(8) $$P_{4mer}\left(t\right)=\frac{Q\left(t\right)}{N},t\in \left\{R_{1}R_{1}R_{1}R_{1},R_{1}R_{1}R_{1}R_{2},\ldots ,R_{7}R_{7}R_{7}R_{7}\right\}$$

where Q(t) is the number of k-mer type t, N is the length of the protein sequence.

### 2.4. Structural Features

Molecular features that rely on lncRNA and protein structure information play a significant role in their interactions. Therefore, we used the secondary structure, hydrogen bonding propensities, and van der Waals interactions to represent the lncRNA’s and protein’s structure information.

For the lncRNA, its secondary structure was obtained through RNAfold [40] based on the minimum free energy algorithm and encoded by replacing each bracket with one and each dot with zero. Meanwhile, we adopted purine and pyrimidine contact information from a set of 41 RNA-protein complexes [41] in lncPro [42] to encode their hydrogen bonding propensities and van der Waals interactions. Each lncRNA structure is represented in these three numerical feature vectors.

For the protein, its secondary structure was obtained through Predator [43], based on its amino acid sequence, and was encoded by replacing each amino acid with the corresponding Chou–Fasman [44] propensity in LncADeep [45]. The hydrogen bonding propensities were encoded by using Grantham propensities [46] and Zimmerman propensities [47]. The Van der Waals interaction was encoded by using the Kyte–Doolittle [48] and Bull–Breese propensities [49]. Each protein structure is represented in these five numerical feature vectors.

However, each lncRNA and protein feature vector is of different dimension, which depends on the length of the corresponding RNA or protein sequence, and the CNN model requires fixed matrix inputs. We adopted the Fourier transform to unify the dimension. The formula of the Fourier series can be expressed as:

(9) $$X_{k}^{\prime }=\sqrt{\frac{2}{L}}\overset{L}{\underset{n=0}{\sum }}X_{n}\cos\left[\frac{\pi }{L}\left(n+\frac{1}{2}\right)\left(k+\frac{1}{2}\right)\right],k=0,1,\ldots ,9$$

where L is the length of the original feature vector.

Here, those criteria in lncPro were adopted that use the first ten terms of the Fourier series as the new numerical feature vector. In this way, we obtain the lncRNA structure feature vector $\;B_{1}=\left[L_{SS},L_{OHB},L_{OVW}\right]$ of dimension 30 and the protein structure feature vector $B_{2}=\left[P_{SS},P_{GHB},P_{ZHB},P_{KVW},P_{BVW}\right]$ of dimension 50.

### 2.5. Convolutional Neural Networks

Convolutional neural network (CNN) is an effective tool in the field of predictive lncRNA–protein interaction [50,51]. Therefore, we introduce CNN as an algorithm to analyze the input raw sequence composition features, hand-designed features and structure features. In this work, the CNN model consists of four modules, including GloCNN, LocCNN, FC, and SS. The architecture of the CNN model and its detailed hyperparameters are shown in Supplementary Figure S1.

For the GloCNN module, the lncRNA and protein encoding matrices were fed into two-layer 1-channel CNNs (convolutional layer, batch normalization, max pooling layer) to extract raw sequence global features, where all the sequences were transformed into fixed-length equivalents. By experimentation, the prediction accuracy did not grow significantly when the layer number was larger than 2, and a larger hidden layer number brought more computation. In addition, a dropout layer was used to accelerate the training process and avoid overfitting. Finally, the lncRNA and protein feature maps were concatenated and their dimensionality reduced to 64 through a fully connected layer.

The LocCNN module used two-layer multi-channel CNNs to extract raw sequence local features, wherein all the sequences had multiple subsequences. The number of channels was determined by the local sequence encoding. After a dropout layer and fully connected layer, the lncRNA and protein feature maps were concatenated and their dimensionality reduced to 32.

For the FC module, the lncRNA and protein hand-designed feature vectors, $A_{1}$ and *A*_2_, were fed into a two-layer fully connected layer to extract high-level features, followed concatenating the two feature maps, from which we extracted useful information through a fully connected layer.

The SS module used a two-layer fully connected layer to analyze the lncRNA and protein structure feature vectors, $B_{1}$ and $B_{2}$. Then, the feature maps were concatenated and further fed into a two-layer fully connected layer to extract useful information and reduce therr dimensionality to 32.

Finally, the feature maps of four basic module outputs were concatenated and further fed into the two fully connected layers to predict the probabilities of lncRNA–protein interactions.

### 2.6. Evaluation Metrics

In this study, we used the metrics of accuracy (ACC), Matthew’s correlation coefficient (MCC), F1_score (F1), sensitivity (SN), specificity (SP), and positive predictive value (PPV) to measure the performance of LGFC-CNN. The formulas of the six measurements are as follows:

(10) $$ACC=\frac{TP+TN}{TP+TN+FP+FN}$$

(11) $$MCC=\frac{TP\times TN-FP\times FN}{\sqrt{\left(TP+FP\right)\left(TP+FN\right)\left(TN+FP\right)\left(TN+FN\right)}}$$

(12) $$F1=2\times \frac{TP}{2TP+FP+FN}$$

(13) $$SN=\frac{TP}{TP+FN}$$

(14) $$SP=\frac{TN}{TN+FP}$$

(15) $$PPV=\frac{TP}{TP+FP}$$

where *TP*, FP, *TN*, FN represent true positive, false positive, true negative, and false negative. Furthermore, we drew the area under the ROC curve (AUC) and the precision-recall curve (PRC) to measure the performance of LGFC-CNN.

## 3. Results

In this section, we first downloaded and ran the algorithms RPISeq-RF [15], RPISeq-SVM [15], LPI-BLS [17], IPMiner [16] following their respective papers and compared the performance of LGFC-CNN with these methods on the benchmark dataset RPI21850. We then compared LGFC-CNN with other methods on the datasets RPI7317 and RPI1847 to test the reliability and robustness of LGFC-CNN. We further used the random forest classifier [24] to compare various lncRNA and protein hand-designed feature combinations and analyze our negative sample strategy’s effect. Finally, we verified the effectiveness of the proposed multi-type feature-combination method and the effect of the hyper-parameters in the CNN model. During the experiment process, we selected 70% of samples from these datasets as the training set, and then randomly selected 50% of the remaining data as the fixed-validation set and the assigned the remaining samples to the test set. 

### 3.1. Performance of LGFC-CNN in Predicting lncRNA–Protein Interactions

To assess the performance of our LGFC-CNN, we first compared LGFC-CNN with RPISeq-RF, RPISeq-SVM, LPI-BLS, and IPMiner on the benchmark dataset RPI21850. The results of LGFC-CNN and the other four methods are shown in Table 2, from which we can see that performance of our LGFC-CNN was superior to the other four methods on the RPI21850 dataset. On the RPI21850 dataset, LGFC-CNN yielded an accuracy of 94.14%, which was 1.8%, 2.04%, 2.73, and 1.84% higher than that of RPISeq-RF, RPISeq-SVM, LPI-BLS, and IPMiner, respectively. The MCC of LGFC-CNN was 0.8853, which was 3.72%, 4.28%, 5.67%, and 3.92% higher than RPISeq-RF, RPISeq-SVM, LPI-BLS, and IPMiner, respectively. The F1-score of LGFC-CNN was 0.9435, which was 1.8%, 2.11%, 2.82%, and 1.97% higher than RPISeq-RF, RPISeq-SVM, LPI-BLS, and IPMiner, respectively. The SN of LGFC-CNN was 97.9%, which was 2.75%, 4%, 5.07%, and 4.55% higher than RPISeq-RF, RPISeq-SVM, LPI-BLS, and IPMiner, respectively. 

**Figure 4:**
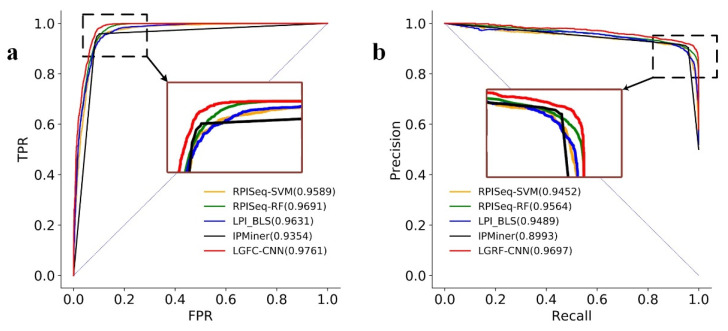
(**a**) The ROC curves and (**b**) the PRC curves of LGFC-CNN and other four methods on the RPI21850 dataset.

The ROC curves and the PRC curves of LGFC-CNN and the other four methods on RPI21850 are shown in Figure 4. From the figure, it can be seen that the AUC score of LGFC-CNN reached 0.9761, which is higher than that of RPISeq-SVM, RPISeq-RF, and IPMiner, respectively. The PRC score of LGFC-CNN reached 0.9697, and its curve can wrap the curves of other methods. These results indicate that LGRF-CNN performs well in predicting lncRNA–protein interactions.

To further test the reliability and robustness of LGRF-CNN, we also comparde LGRF-CNN with four comparison methods on the RPI7317 and RPI1847 datasets. Table 3 lists the results of LGRF-CNN, RPISeq-RF, RPISeq-SVM, LPI-BLS, and IPMiner on these two datasets. On dataset RPI7317, the ACC of LGRF-CNN was 92.94%, which was better than RPISeq-RF (ACC: 90.98%), RPISeq-SVM (ACC: 91.53%), LPI-BLS (ACC: 91.44%), IPMiner (ACC: 91.34%), and showed specific improvement in the other five indicators. On dataset RPI1847, the ACC of LGRF-CNN was 98.19%, which is better than RPISeq-RF (ACC: 96.21%), RPISeq-SVM (ACC: 95.85%), LPI-BLS (ACC: 96.75%), IPMiner (ACC: 96.39%). 

**Figure 5:**
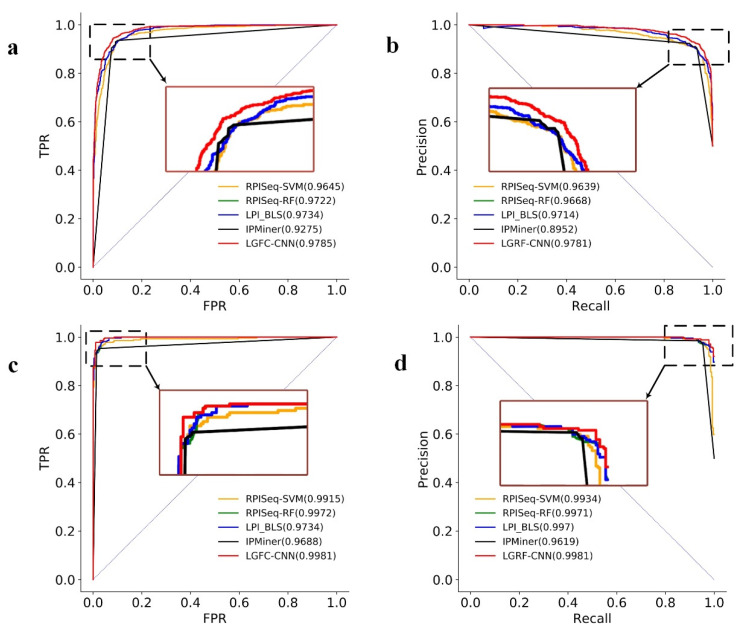
(**a**) The ROC curves and (**b**) the PRC curves of LGFC-CNN and other four methods on RPI7317. (**c**) The ROC curves and (**d**) the PRC curves of LGFC-CNN and other four methods on RPI1847.

Figure 5 shows the ROC curves and PRC curves of LGRF-CNN and other methods on RPI7317 and RPI1847. The figure shows that the AUC scores of LGRF-CNN on RPI7317 and RPI1847 are 0.9785 and 0.9981, respectively, and the PRC scores reach 0.9781 and 0.9981, respectively. The curves of LGFC-CNN can wrap the curves of other methods. The above results show that different data sources will affect the performance of LGFC-CNN, but it can still maintain superior performance.

### 3.2. Performance Comparison between Different Feature Combinations in Predicting lncRNA–Protein Interactions

**Figure 6:**
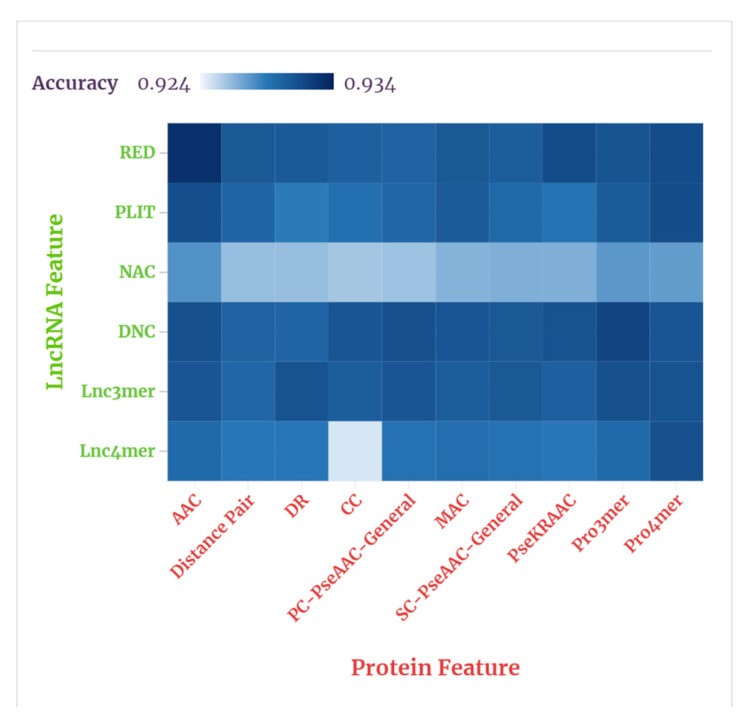
The heat map generated from the feature combinations in RPI21850.

Before feeding the hand-designed feature vectors into the FC module, we needed to select the top three features of superior predictive effect to represent the lncRNA and protein. Six features of the lncRNA were combined with ten features of the protein and each feature combination was ranked according to their individual average performances in the random forest classifier. For ranking the feature combinations, we compared the ACC of each combination to predict the lncRNA–protein interactions in RPI21850, and the results obtained through experiments are shown in supplementary Tables S1–S10. Figure 6 shows the results, visually, through a heat map.

As shown in our tables and figures, the average prediction accuracies of the combinations of $L_{RED}$, $L_{PLIT}$, $L_{NAC}$, $L_{DNC}$, *L*_3*mer*_, $L_{4mer}$ and ten protein features were 93.1%, 93.01%, 92.67%, 93.09%, 93.07%, 92.9%, respectively. The average prediction accuracies of the combinations of $P_{AAC}$, $P_{Dis}$, $P_{DR}$, $P_{CC}$, $P_{PC-PseAAC}$, $P_{MAC}$, $P_{SC-PseAAC}$, *P_PseKRAAC_*, $P_{3mer}$, $P_{4mer}\;$ and six lncRNA features were 93.07, 92.92%, 92.86%, 92.95%, 92.98%, 92.96%, 93.03%, 93.06%, 93.1%, 93.06%, also respectively. Accordingly, the three lncRNA features $L_{red}$, *L*_*dnc*_, and $L_{3mer}$ were selected to represent the lncRNA, and the three protein features $P_{AAC}$, $P_{3mer}$, and *P*_4*mer*_ were selected to represent the protein. These results indicate that the features related to coding potentials and physicochemical properties appear to be more suitable for predicting lncRNA–protein interactions.

### 3.3. Comparison between Four Modules of LGFC-CNN

**Figure 7:**
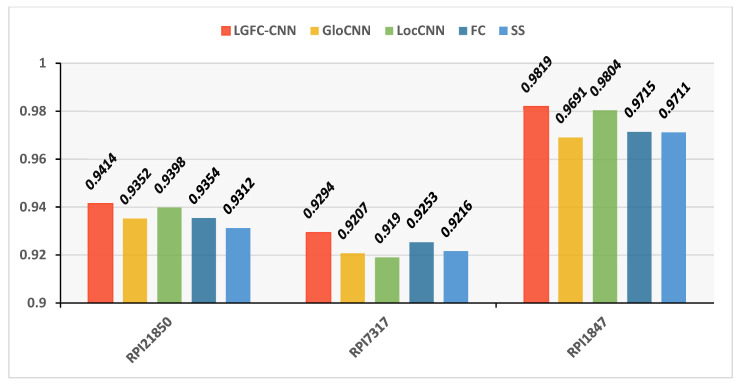
The performance of LGFC-CNN and four different basic modules.

Our LGFC-CNN model contains four fundamental modules: a LocCNN module, a GloCNN module, an FC module, and an SS module. To investigate the superiority of LGFC-CNN, we compared LGFC-CNN with four basic modules on datasets RPI21850, RPI7317, and RPI1847. The performance of LGFC-CNN and four different basic modules are shown in a histogram Figure 7.

The compared results show that, on the benchmark dataset RPI21850, the prediction accuracy of LocCNN module was 93.98%, which was higher than the 93.52% of the GloCNN module, the 93.54% of the FC module, and the 93.12% of the SS module. On PRI7317, the prediction accuracy of the FC module was 92.53%, which was higher than the 92.07% of the GloCNN module, the 91.9% of the LocCNN module, and the 92.16% of the SS module. On the dataset PRI1847, the prediction accuracy of LocCNN module was 98.04%, which was higher than the 96.91% of GloCNN module, the 97.15% of the FC module, and the 97.11% of the SS module. It can be seen from the results that the prediction accuracy of any single module could always exceed that of the other modules, and none of them was as good as the overall LGFC-CNN. It shows that the LGFC-CNN model we have proposed has the advantages of the four basic prediction modules. The combination of global sequence features, local sequence features, hand-designed features, and structural features can provide more comprehensive lncRNA–protein prediction results. 

### 3.4. Effectiveness of Selecting a Negative Sample Strategy

To show the effectiveness of our strategy for selecting negative samples, we first constructed three new datasets, ranRPI21850, ranRPI7317, and ranRPI1847, by randomly pairing the lncRNA and protein and removing the duplicate interaction pairs. ranRPI21850 contained 21,850 lncRNA–protein interaction pairs and 21,850 pairs of lncRNA–protein non-interaction pairs; ranRPI7317 contained 7317 lncRNA–protein interaction pairs and 7317 pairs of lncRNA–protein non-interaction pairs; and ranRPI1847 contained 1847 lncRNA–protein pairs. Then we predicted the lncRNA–protein interactions in RPI21850, RPI7317, RPI1847, ranRPI21850, ranRPI7317, and ranRPI1847 under the same conditions. Table 4 shows the effect of LGFC-CNN on three datasets generated by our negative sample-generation strategy and random pair generation strategy.

It can be seen from Table 4 that the accuracy of LGFC-CNN on RPI21850 was 94.14%, which was 2.59% higher than that on ranRPI21850. The accuracy on RPI7317 was 92.94%, which was 2.96% higher than that on ranRPI7317. The accuracy on RPI1847 was 98.19%, which was 1.44% higher than that on ranPRI1847. These results show that the strategy of selecting negative samples used in this work is effective and can improve the prediction performance for lncRNA–protein interactions. 

### 3.5. Effects of Hyper-Parameters in LGFC-CNN

Our LGFC-CNN model consists of four basic modules for analyzing global sequence features, local sequence features, hand-designed features, and structure features. To inves-tigate how the hyper-parameters of the convolutional layer kernel number and the fully connected layer neuron number affect the performance of LGFC-CNN, we change one pa-rameter value at a time by fixing other parameters on the validation set of RPI21850 to im-plement our LGFC-CNN. For the GloCNN module, Kernel-G of the convolutional layer was set as n × 10, n × 20, n × 30, n × 40, n × 50 (n for lncRNA is 4, n for protein is 7). For the LocCNN module, Kernel-L of the convolutional layer was set as n × 10, n × 20, n × 30, n × 40, n × 50 (n for lncRNA is 4, n for protein is 7). The neuron number of the four fully connected layers Dense-G, Dense-L, Dense-FC, and Dense-SS was set to 16, 32, 48, and 64, respec-tively. 

Supplementary Tables S11–S16 shows shows the results of LGFC-CNN using different hy-per-parameters on the validation set of RPI21850, from which we can find that the hy-per-parameters have some influence on the prediction results. When setting the kernel-G as n × 40, kernel-L as n × 30, Dense-G as 64, Dense-L as 32, Dense-FC as 32, and Dense-SS as 32, LGFC-CNN achieves the best performance.

## 4. Discussion

In this study, we proposed a novel method based on deep learning and using multiple types of features to predict lncRNA–protein interactions. On the benchmark dataset we constructed, LGFC-CNN achieved an accuracy of 94.14%, an MCC of 0.8853, an F1-score of 0.9435, an SN of 97.9%, an SP of 90.39%, and a PPV of 91.06%. The experimental results on RPI7317 and RPI1847 also showed the effectiveness of LGFC-CNN.

LGFC-CNN had superior performance in predicting lncRNA–protein interactions; we believe that there are several reasons why. Firstly, for the structure of the negative sample strategy, our method considers that if protein $p^{\prime }$ is dissimilar to q, there is a low possibility that $p^{\prime }$ interacts r [27], and we also consider that there is a certain probability that lncRNA is related to those proteins with higher scores. The experimental results verify that our negative sample strategy is more effective than the commonly used random pairing method. Secondly, in the raw sequence features, we used both global sequence features and local sequence features. Our method considers the global sequence’s overall characteristics and considers the critical role of the local sequence in the lncRNA–protein interaction. Thirdly, we compare the combinations of various types of lncRNA features and protein features in terms of hand-designed features and use secondary features, hydrogen bonding propensities and van der Waals interactions as structure features. Finally, the combination of global sequence features, local sequence features, hand-designed features, and structure features can provide more comprehensive lncRNA-protein prediction results.

Although LGFC-CNN achieves better performance in predicting lncRNA–protein interactions, there are still limitations. On the one hand, since most of the high-quality experimentally verified human lncRNA–protein pairs are mainly derived from the NPInter dataset [18], our method can still only be trained on a few datasets. When there are multiple data sources, deep learning can play a more significant role, so more data sources are needed to cover more possible situations. On the other hand, there are many types of hand-designed and structural features, and what we have here-explored is only part of them. Finding better hand-designed and structural features and exploring better network structures to improve the hand-designing of feature and structure modules’ performance will be the focus of our future work. In future work, we will explore the effect of LGFC-CNN in the interaction between lncRNA with miRNA and hope to find a suitable method that can integrate the LPI similarity network, so that more types of features can be used to improve the classification effect.

## 5. Conclusions

To understand the various regulatory mechanisms and pathogenic mechanisms involved in lncRNA through lncRNA–protein interactions, some computed methods were developed for predicting lncRNA–protein interactions [11,12,13]. However, there is currently no method to combine raw sequence features, hand-designed features and structural features to predict lncRNA–protein interactions. In this work, we presented a novel deep learning method, LGFC-CNN, to predict lncRNA–protein interactions by using multiple types of features. We introduced two sequence preprocessing methods to transform arbitrarily long sequences into fixed-length sequences and feed them into two modules to gain raw global and local sequence features. Meanwhile, we selected hand-designed features by comparing the predictive effects of different lncRNA and protein feature combinations. Furthermore, we obtained lncRNA and protein structural features and unified their dimensions through a Fourier transform. The experimental results show that our LGFC-CNN had a better performance than other latest methods. All in all, LGFC-CNN is a feasible and effective tool for predicting human lncRNA–protein interactions. Our LGFC-CNN model combines raw sequence features, hand-designed features and structure features is also a potential tool for the other bioinformatics classification tasks.

## Figures and Tables

**Table 1:** Numeric description of the datasets.

Dataset	lncRNAs	Proteins	Interaction Pairs	Non-Interaction Pairs
RPI21850	4221	701	21850	21850
RPI7317	1874	118	7317	7317
RPI1847	1939	60	1847	1847

**Table 2:** The result of LGFC-CNN and other four methods on the RPI21850 dataset.

Methods	ACC	MCC	F1-Score	SN	SP	PPV
LGFC-CNN	**0.9414**	**0.8853**	**0.9435**	**0.979**	0.9039	0.9106
RPISeq-RF	0.9234	0.8481	0.9255	0.9515	0.8954	0.9009
RPISeq-SVM	0.921	0.8425	0.9224	0.939	0.903	0.9063
LPI-BLS	0.9141	0.8286	0.9153	0.9283	0.8999	0.9027
IPMiner	0.923	0.8461	0.9238	0.9335	**0.9124**	**0.9142**

**Table 3:** The result of LGFC-CNN and other four methods on the RPI7317 and RPI1847 datasets.

RPI7317						
Methods	ACC	MCC	F1-Score	SN	SP	PPV
LGFC-CNN	**0.9294**	**0.8589**	**0.9299**	**0.9371**	**0.9217**	**0.922**
RPISeq-RF	0.9098	0.8202	0.9116	0.9299	0.8897	0.894
RPISeq-SVM	0.9153	0.8311	0.9169	0.9344	0.8961	0.9
LPI-BLS	0.9144	0.8288	0.9151	0.9226	0.9061	0.9077
IPMiner	0.9134	0.8269	0.9139	0.918	0.9088	0.9097
RPI1847						
LGFC-CNN	**0.9819**	**0.964**	**0.9818**	0.9747	0.9856	0.989
RPISeq-RF	0.9621	0.9243	0.9617	0.9531	0.9711	0.9706
RPISeq-SVM	0.9585	0.9191	0.957	0.9242	**0.9928**	**0.9922**
LPI-BLS	0.9675	0.9352	0.9672	0.9567	0.9783	0.9779
IPMiner	0.9639	0.9287	0.9631	0.9422	0.9856	0.9849

**Table 4:** Results of LGFC-CNN on six datasets generated by different negative sample generation strategy.

Datasets	ACC	MCC	F1-Score	SN	SP	PPV
RPI21850	**0.9414**	**0.8853**	**0.9435**	0.979	**0.9039**	**0.9106**
ranRPI21850	0.9155	0.8381	0.9207	**0.9805**	0.8505	0.8677
RPI7317	**0.9294**	**0.8589**	**0.9299**	**0.9371**	**0.9217**	**0.9228**
ranRPI7317	0.8998	0.7996	0.9003	0.9052	0.8944	0.8954
RPI1847	**0.9819**	**0.964**	**0.9818**	**0.9747**	**0.9856**	**0.989**
ranRPI1847	0.9675	0.9359	0.9682	**0.9892**	0.9458	0.9481

## Data Availability

The source code and datasets for this work can be obtained from https://github.com/consen1/LGFC-CNN (accessed on 20 June 2021).

## Supplementary Materials

The following are available online at https://www.mdpi.com/article/10.3390/genes12111689/s1. The explanations of the other three lncRNA and seven protein feature encodings we use for comparison. Figure S1: The architecture of the CNN model; Tables S1–S10: The tables of six lncRNA feature and ten protein feature combinations; Tables S11–S16: The tables of LGFC-CNN using different hyper-parameters on validation set of RPI21850.
