# Supplementary material for: LGFC-CNN: Prediction of lncRNA-Protein Interactions by Using Multiple Types of Features through Deep Learning
Source: Genes (Basel). 2021 Oct 24;12(11):1689. doi: 10.3390/genes12111689 (PMC8621699; doi:10.3390/genes12111689)
Supplement: Supplementary file 1 [file genes-12-01689-s001.zip › Supplementary S-1.pdf]

Supplementary File S-1

1. The other three lncRNA feature encodings

1.1. lncRNA feature PLIT

PLIT is a new tool which uses L1 regularization for feature selection and a Random Forest classifier for classification of sequences. The tool determined 10 essential features including structures, sequences, expression profiles and histone modification signals. Therefore, we use L_{PLIT} to represent the features generated by PLIT.

1.2. lncRNA feature NAC

The Nucleic Acid Composition (NAC) encoding calculates the frequency of each nucleic acid type in a nucleotide sequence. The frequencies of all 4 natural nucleic acids can be calculated as:

$$L_{NAC}(t) = \frac{N(t)}{N}, t \in \{A, C, G, T\}$$

where N(t) is the number of nucleic acid type t, while N is the length of a nucleotide sequence.

1.3. lncRNA feature 4-mer

L_{4mer} represents the normalized occurrence frequencies of 4 neighboring base pairs in the RNA sequence, which has been successfully applied to human gene regulatory sequence prediction and enhancer identification. It can be computed as follows:

$$L_{3mer}(t) = \frac{M(t)}{N}, t \in \{AAAA, AAAT, AAAC, \ldots, GGGG\}$$

where $M(t)$ is the number of k-mer type t, N is the length of a nucleotide sequence.

2. The other seven protein feature encodings

2.1. Protein feature Distance Pair

P_{Dis} can incorporate more sequence-order information by gradually increasing the value of integer d. It can be formulated by:

$$f(R_i, R_j|d)$$

where R_i and R_j can be any of the 20 native amino acids in a protein chain, and d represents the distance counted by the number of amino acids between R_i and R_j along the protein chain.

2.2. Protein feature Distance-based Residue

P_{DR} is similar as the Distance-based Top-1-gram approach (DT), except that the native protein sequence was directly converted into the feature vector without replacing the amino acids with Top-1-grams.

2.3. Protein feature Cross covariance

P_{CC} variable measures the correlation of two different properties between two residues separated by lg along the sequence, which can be calculated by:

$$CC(i1, i2, lg) = \sum_{j=1}^{L-lg} \left(S_{i1,j} - \overline{S}_{i1}\right)\left(S_{i2,j+lg} - \overline{S}_{i2}\right)/(L - lg)$$

where i1, i2 are two different amino acids and $\overline{S}_{i1}$ ($\overline{S}_{i2}$) is the average score for amino acid i1 (i2) along the sequence. Since the CC variables are not symmetric, the total number of CC variables is 380*LG.

2.4. Protein feature PC-PseAAC-General

$P_{PC-PseAAC}$ was originally introduced to represent protein samples for improving protein subcellular localization prediction and membrane protein type prediction. Like the vanilla

amino acid composition (AAC) method, it characterizes the protein mainly using a matrix of amino-acid frequencies, which helps with dealing with proteins without significant sequential homology to other proteins. Compared to AAC, additional information is also included in the matrix to represent some local features, such as correlation between residues of a certain distance.

2.5. Protein feature Moran autocorrelation

P_{MAC} is demonstrated that protein α-helix content can be predicted from an autocorrelation analysis of the protein hydrophobicity sequence. The Fourier transform of the autocorrelation function yields the spectral densities or weights of the various frequencies contributing to the autocorrelation function. Using sequence and secondary structure data from more than 160 proteins and domains, a linear relationship was found between spectral density at periodicity 3.7 and protein α-helix content (r = 0.83).

2.6. Protein feature SC-PseAAC-General

$P_{SC-PseAAC}$ is introduced to represent the statistical sample of a protein. The novel representation contains 20 + 2λ discrete numbers: the first 20 numbers are the components of the conventional amino acid composition; the next 2λ numbers are a set of correlation factors that reflect different hydrophobicity and hydrophilicity distribution patterns along a protein chain.

2.7. Protein feature PseKRAAC

$P_{PseKRAAC}$ delivers more capability for protein research by incorporating three crucial parameters that describes protein composition. By implementing reduced amino acid alphabets, the protein complexity can be significantly simplified, which leads to decrease chance of overfitting, lower computational handicap and reduce information redundancy.
