# Supplementary material for: LGFC-CNN: Prediction of lncRNA-Protein Interactions by Using Multiple Types of Features through Deep Learning
Source: Genes (Basel). 2021 Oct 24;12(11):1689. doi: 10.3390/genes12111689 (PMC8621699; doi:10.3390/genes12111689)
Supplement: Supplementary file 1 [file genes-12-01689-s001.zip › Supplementary S-2.pdf]

Supplementary File S-2

1. The architecture of the CNN model

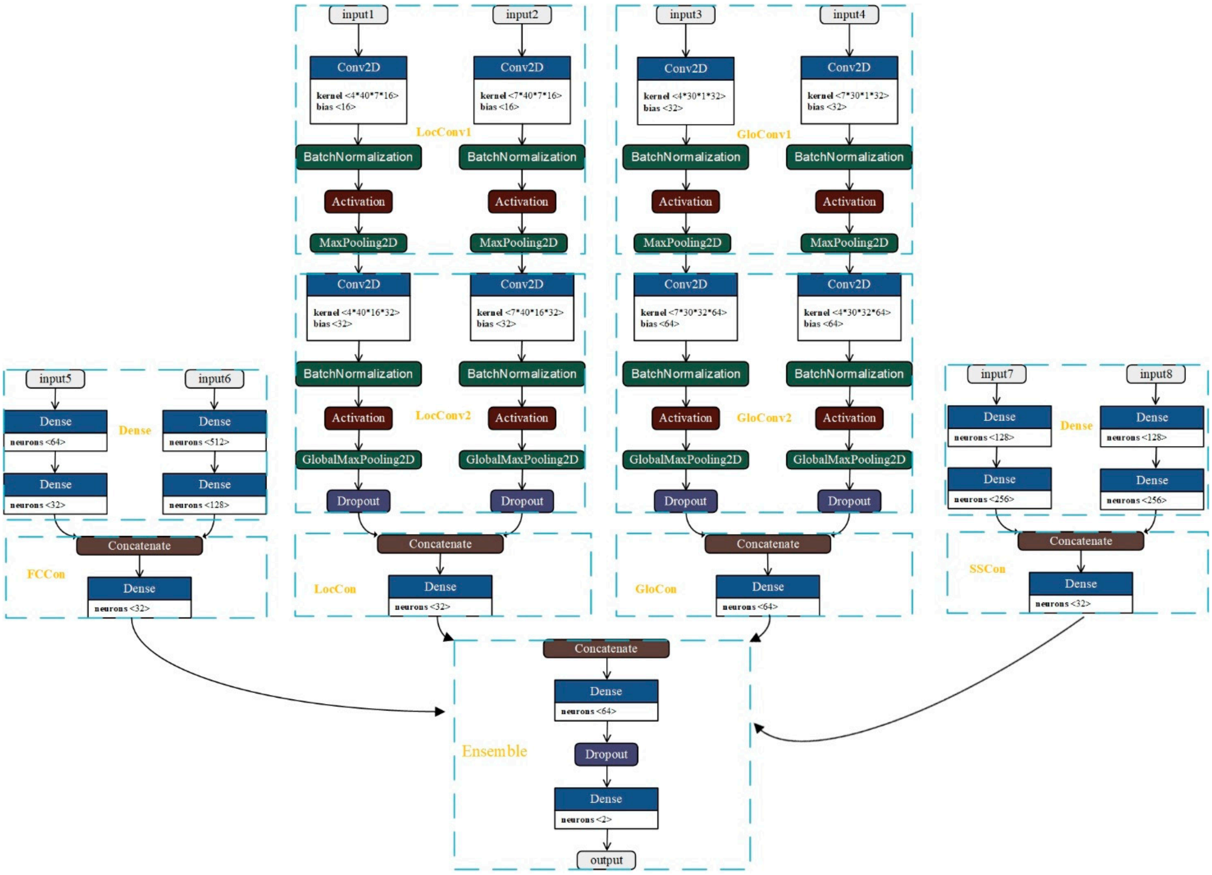

Figure S1. The architecture of the CNN model.

Input1 and input2 are the inputs of the GloCNN module, input3 and input4 are the inputs of the LocCNN module, input5 and input6 are the FC module inputs, input7 and input8 are the SS module inputs. Conv2D is a convolution layer, Batch Normalization is a batch normalization layer, Activation is a Relu activation layer, Maxpooling2D is a pooling layer using the max-pooling scheme, GlobalMaxPooling2d is a pooling layer using the global max-pooling scheme, Dropout is a dropout layer, Dense is a fully connected layer, Concatenate is a concatenate layer.

2. The tables of six lncRNA feature and ten protein feature combinations

Table S1. The result of protein feature AAC and the six lncRNA feature combinations

lncRNA Features	ACC	MCC	F1-score	SN	SP	PPV
RED	0.9332	0.8676	0.9349	0.96	0.9063	0.9111
PLIT	0.9314	0.8639	0.9331	0.9576	0.9051	0.9099
NAC	0.9278	0.8567	0.9296	0.9521	0.9036	0.9081
DNC	0.9314	0.8643	0.9334	0.9616	0.9011	0.9068
Lnc3mer	0.9315	0.8644	0.9334	0.9597	0.9033	0.9085
Lnc4mer	0.9292	0.8599	0.9313	0.9588	0.8996	0.9052

Table S2. The result of protein feature Distance Pair and the six lncRNA feature combinations

lncRNA Features	ACC	MCC	F1-score	SN	SP	PPV
RED	0.9307	0.8628	0.9326	0.9582	0.9033	0.9083
PLIT	0.93	0.8614	0.932	0.9591	0.9008	0.9063
NAC	0.9262	0.8532	0.9278	0.9484	0.9039	0.908
DNC	0.9301	0.8617	0.9321	0.9591	0.9011	0.9066
Lnc3mer	0.9298	0.861	0.9317	0.9576	0.902	0.9072
Lnc4mer	0.9289	0.8593	0.9309	0.9582	0.8996	0.9052

Table S3. The result of protein feature DR and the six lncRNA feature combinations

lncRNA Features	ACC	MCC	F1-score	SN	SP	PPV
RED	0.9306	0.862	0.9321	0.953	0.9081	0.9121
PLIT	0.9288	0.8584	0.9304	0.9521	0.9054	0.9096
NAC	0.9262	0.8533	0.9279	0.95	0.9023	0.9068
DNC	0.93	0.8613	0.9319	0.9582	0.9017	0.907
Lnc3mer	0.931	0.8631	0.9327	0.9555	0.9066	0.911
Lnc4mer	0.9289	0.8589	0.9307	0.9542	0.9036	0.9082

Table S4. The result of protein featurn CC and the six lncRNA feature combinations

lncRNA Features	ACC	MCC	F1-score	SN	SP	PPV
RED	0.9303	0.862	0.9322	0.9588	0.9017	0.9071
PLIT	0.9294	0.8598	0.9311	0.9545	0.9042	0.9088
NAC	0.9259	0.8526	0.9275	0.9484	0.9033	0.9075
DNC	0.9309	0.8634	0.933	0.9616	0.9002	0.906
Lnc3mer	0.9304	0.862	0.9322	0.9561	0.9048	0.9095
Lnc4mer	0.9246	0.8506	0.9267	0.953	0.8962	0.9018

Table S5. The result of protein feature PC-PseACC-General and the six lncRNA feature combinations

lncRNA Features	ACC	MCC	F1-score	SN	SP	PPV
RED	0.9301	0.8613	0.9318	0.9542	0.906	0.9104
PLIT	0.9298	0.861	0.9317	0.9576	0.902	0.9072
NAC	0.926	0.8528	0.9276	0.9475	0.9045	0.9085
DNC	0.9309	0.8634	0.9329	0.9613	0.9005	0.9062
Lnc3mer	0.9304	0.8622	0.9323	0.9585	0.9023	0.9076
Lnc4mer	0.9292	0.8599	0.9313	0.9588	0.8996	0.9052

Table S6. The result of protein feature MAC and the six lncRNA feature combinations

lncRNA Features	ACC	MCC	F1-score	SN	SP	PPV
RED	0.9307	0.8629	0.9327	0.9594	0.902	0.9074
PLIT	0.9306	0.8665	0.9344	0.9603	0.9048	0.9098

NAC	0.9266	0.8541	0.9282	0.9491	0.9042	0.9083
DNC	0.9312	0.8639	0.9332	0.961	0.9014	0.907
Lnc3mer	0.9309	0.8631	0.9327	0.9582	0.9036	0.9086
Lnc4mer	0.9294	0.8602	0.9314	0.9582	0.9005	0.906

Table S7. The result of protein feature SC-PseACC-General and the six lncRNA feature combinations

lncRNA Features	ACC	MCC	F1-score	SN	SP	PPV
RED	0.9304	0.8618	0.9321	0.9542	0.9066	0.9109
PLIT	0.9297	0.8606	0.9315	0.9567	0.9027	0.9077
NAC	0.9268	0.8545	0.9285	0.9503	0.9033	0.9076
DNC	0.9307	0.8630	0.9327	0.9603	0.9011	0.9067
Lnc3mer	0.9307	0.8627	0.9325	0.9573	0.9042	0.9090
Lnc4mer	0.9297	0.8608	0.9317	0.9585	0.9008	0.9063

Table S8. The result of protein feature PseKRAAC and the six lncRNA feature combinations

lncRNA Features	ACC	MCC	F1-score	SN	SP	PPV
RED	0.9315	0.8646	0.9335	0.9619	0.9011	0.9068
PLIT	0.9291	0.8595	0.931	0.9576	0.9005	0.9059
NAC	0.9268	0.8546	0.9285	0.9512	0.9023	0.9069
DNC	0.931	0.8637	0.9331	0.9616	0.9005	0.9063
Lnc3mer	0.9303	0.8619	0.9322	0.9585	0.902	0.9073
Lnc4mer	0.9289	0.8595	0.931	0.9597	0.8981	0.904

Table S9. The result of protein feature Pro3mer and the six lncRNA feature combinations

lncRNA Features	ACC	MCC	F1-score	SN	SP	PPV
RED	0.931	0.8636	0.9331	0.961	0.9011	0.9067
PLIT	0.9305	0.8605	0.9315	0.9585	0.9005	0.906
NAC	0.9276	0.8542	0.9283	0.95	0.9033	0.9076
DNC	0.932	0.8653	0.9339	0.9606	0.9033	0.9085
Lnc3mer	0.9312	0.8638	0.9331	0.9594	0.903	0.9082
Lnc4mer	0.9297	0.8608	0.9317	0.9591	0.9002	0.9058

Table S10. The result of protein feature Pro4mer and the six lncRNA feature combinations

lncRNA Features	ACC	MCC	F1-score	SN	SP	PPV
RED	0.9315	0.8646	0.9336	0.9622	0.9008	0.9066
PLIT	0.9314	0.8621	0.9323	0.9573	0.9036	0.9085
NAC	0.9275	0.8538	0.9281	0.9491	0.9039	0.9081
DNC	0.9309	0.8633	0.9329	0.9606	0.9011	0.9067
Lnc3mer	0.931	0.8635	0.933	0.9597	0.9023	0.9077
Lnc4mer	0.9312	0.8639	0.9332	0.9606	0.9017	0.9072

3. The tables of LGFC-CNN using different hyper-parameters on validation set of RPI21850

Table S11. Results of LGFC-CNN on validation set of RPI21850 with different kernel-L size and n*30 kernel-G, 32 neurons in Dense-L, 64 in neurons Dense-G, 32 in neurons Dense-FC, and 32 in neurons Dense-SS

Size of kernel-L	ACC	MCC	F1-score	SN	SP	PPV
n*10	0.9368	0.8776	0.9397	0.9841	0.8895	0.8991
n*20	0.9408	**0.8853**	0.9434	**0.9863**	0.8953	0.9041
n*30	0.9387	0.8788	0.9404	0.9674	**0.91**	**0.9149**
**n*40**	**0.9414**	**0.8853**	**0.9435**	0.979	0.9039	0.9106
N*50	0.9384	0.8784	0.9402	0.9695	0.9072	0.9127

Table S12. Results of LGFC-CNN on validation set of RPI21850 with different kernel-G size and n*40 kernel-L, 32 neurons in Dense-L, 64 in neurons Dense-G, 32 in neurons Dense-FC, and 64 in neurons Dense-SS

Size of kernel-G	ACC	MCC	F1-score	SN	SP	PPV
n*10	0.9385	0.88	0.941	**0.9796**	0.8975	0.9053
n*20	0.9376	0.8769	0.9395	0.9689	0.9063	0.9119
**n*30**	**0.9414**	**0.8853**	**0.9435**	0.979	0.9039	0.9106
n*40	0.9387	0.881	0.9414	0.9841	0.8932	0.9021
N*50	0.939	0.8794	0.9407	0.9674	**0.9106**	**0.9154**

Table S13. Results of LGFC-CNN on validation set of RPI21850 with different Dense-L size and n*40 kernel-L, n*30 kernel-G, 64 in neurons Dense-G, 32 in neurons Dense-FC, and 64 in neurons Dense-SS

Numbers of neurons	ACC	MCC	F1-score	SN	SP	PPV
16	0.9402	0.8837	0.9427	**0.9835**	0.8969	0.9051
**32**	**0.9414**	**0.8853**	**0.9435**	0.979	0.9039	0.9106
48	0.9408	0.8836	0.9428	0.9747	**0.9069**	**0.9129**
64	0.9382	0.8794	0.9407	0.9793	0.8972	0.905

Table S14. Results of LGFC-CNN on validation set of RPI21850 with different Dense-G size and n*40 kernel-L, n*30 kernel-G, 32 in neurons Dense-L, 32 in neurons Dense-FC, and 64 in neurons Dense-SS

Numbers of neurons	ACC	MCC	F1-score	SN	SP	PPV
16	0.9396	0.8813	0.9416	0.9741	**0.9051**	**0.9112**
32	0.9399	0.8824	0.9421	0.9783	0.9014	0.9085
48	0.9399	0.883	0.9424	**0.9826**	0.8972	0.9053
**64**	**0.9414**	**0.8853**	**0.9435**	0.979	0.9039	0.9106

Table S15. Results of LGFC-CNN on validation set of RPI21850 with different Dense-FC size and n*40 kernel-L, n*30 kernel-G, 32 in neurons Dense-L, 64 in neurons Dense-G, and 64 in neurons Dense-SS

Numbers of neurons	ACC	MCC	F1-score	SN	SP	PPV
16	0.941	0.884	0.9429	0.9753	0.9067	0.9126
**32**	**0.9414**	**0.8853**	**0.9435**	**0.979**	0.9039	0.9106
48	0.9388	0.8797	0.9408	0.9728	0.9048	0.9109
64	0.9404	0.8825	0.9422	0.9719	**0.9088**	**0.9142**

Table S16. Results of LGFC-CNN on validation set of RPI21850 with different Dense-SS size and n*40 kernel-L, n*30 kernel-G, 32 in neurons Dense-L, 64 in neurons Dense-G, and 32 in neurons Dense-FC

Numbers of neurons	ACC	MCC	F1-score	SN	SP	PPV
16	0.9388	0.8803	0.9411	0.9774	0.9002	0.9074
32	0.9414	0.8853	0.9435	0.979	0.9039	0.9106
48	0.9371	0.8789	0.9402	0.9887	0.8856	0.8963
64	0.9355	0.8717	0.9368	0.9561	0.9149	0.9183
